# Association of Nasal Nostril Stenosis with Bilateral Choanal Atresia: A Case Report

**Published:** 2014-01

**Authors:** Shahin Abdollahifakhim, Mehrnoush Mousaviagdas

**Affiliations:** 1 *Departments of Otorhinolaryngolog, Tabriz University of Medical Sciences, Tabriz, Iran.*

**Keywords:** Choanal atresia, Nostril stenosis, Rare case

## Abstract

**Introduction:**

Neonatal nasal airway obstruction induces various degrees of respiratory distress. The management of this disease, including surgical repair, will depend on the severity and location of the obstruction. We describe here a case of congenital nasal nostril stenosis that required surgical repair for stenting of both nares after coanal atresia repair.

**Case Report:**

A 2 days old female newborn referred to neonatal department of Tabriz Children’s Hospital affiliated to the University of Medical Sciences of Tabriz, Iran on the 3rd of December, 2011 immediately after birth with respiratory distress due to bilateral coanal atresia and nasal hypoplasia with very small nostrils. CT scan showed normal brain and bilateral choanal atresia with normal size Pyriform apertures.

**Conclusion:**

Nasal obstruction can lead to airway compromise and respiratory distress. Congenital bony nasal deformities are being recognized as an important cause of newborn airway obstruction. Nasal hypoplasia is seen in many craniofacial syndromes. Although our patient had hypoplastic nostrils with respiratory distress due to bilateral coanal atresia, correction of hypoplastic nostrils was necessary for completing the operation of choanal atresia.

## Introduction

Neonatal nasal airway obstruction induces various degrees of respiratory distress (1). The management of this disease, including surgical repair, will depend on the severity and location of the obstruction (2). Here, we describe a case of congenital nasal nostril stenosis that required surgical repair for stenting of both nares after coanal atresia repair.

## Case Report

**Fig1:**
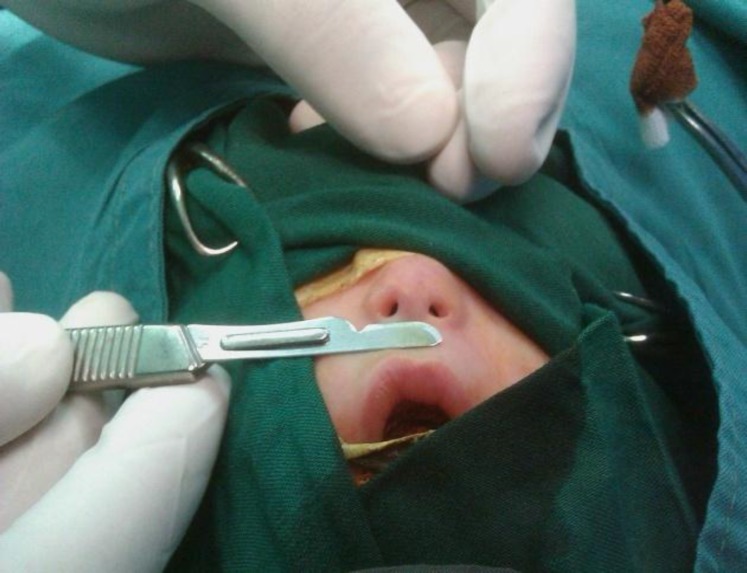
Nasal hypoplasia with very small nostrils

**Fig2:**
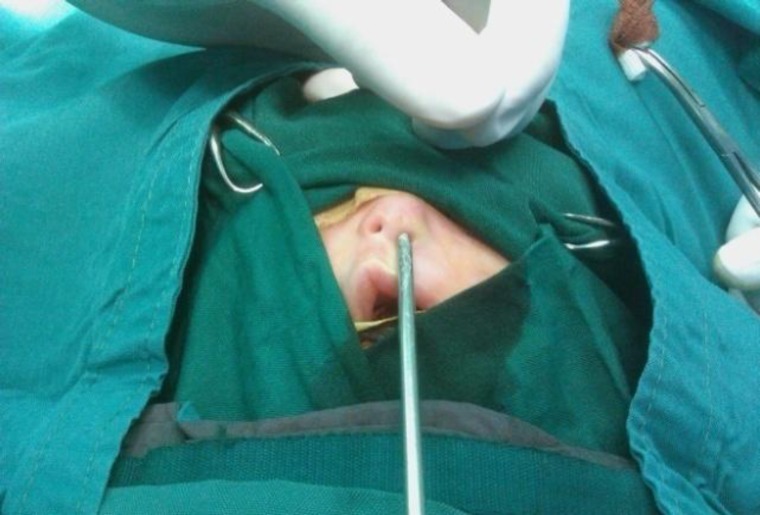
Trans-nasal dilation catheters couldn’t pass through the nostrils

A 2 days old female newborn was referred to the neonatal department of Tabriz Children’s Hospital affiliated to the University of Medical Sciences of Tabriz, Iran on the 3^rd^ of December 2011, immediately after birth with respiratory distress due to bilateral coanal atresia and nasal hypoplasia with very small nostrils (Fig.1). Her respiratory distress improved with oral airway. Her gestational age was 38 weeks. Birth weight was 3000 g and birth length was 45 cm. She was the first child of unrelated parents. The mother and father were 21and 26 years old, respectively. In the physical exam we noticed that the feeding tube sized with trans-nasal dilation catheters couldn’t pass through the nostrils (Fig. 2). 

**Fig 3:**
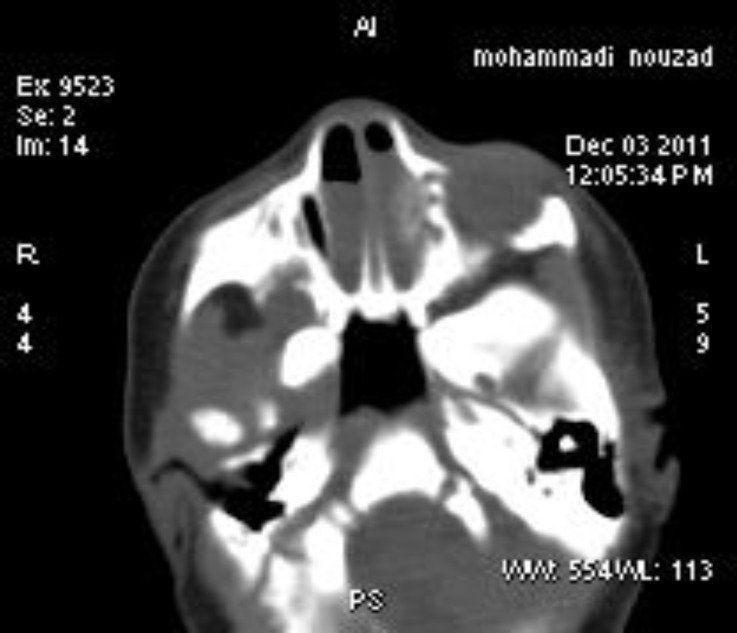
CT- Scan of bilateral choanal atresia

The small feeding tube failed to pass through the posterior choans. CT scan showed normal brain and bilateral choanal atresia. Pyriform apertures were normal in size. Nasal cavities except for anterior nares were normal. There wasn’t any evidence of mid-nasal stenosis (Fig.3). Patient’s evaluation for other nomalies (Cardiac, Kidney, brain, skeletal and hearing) didn’t show any abnormalities.

**Fig 4:**
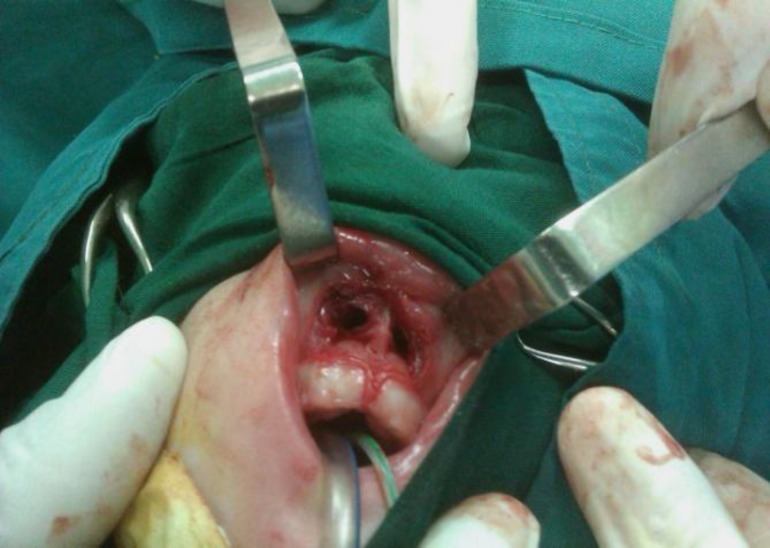
Sublabial approach for choanal atresia

**Fig 5:**
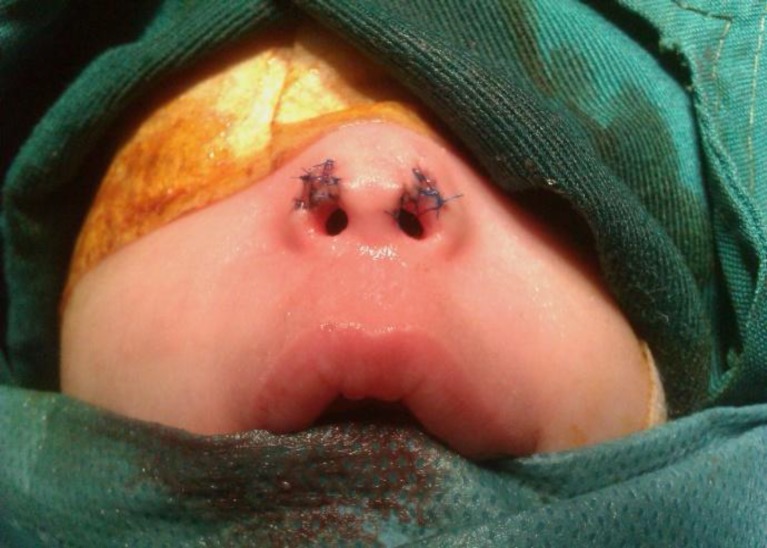
Z plasty for anterior nasal stenosis

**Fig 6:**
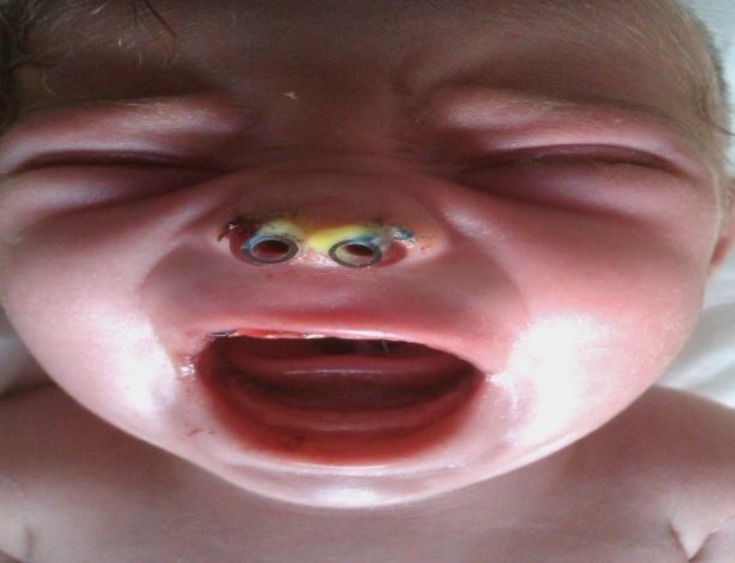
Postoperative photo with endotracheal tubes fixed sublabially

Because of severe anterior nasal stenosis it was impossible to open the atretic plate transnasally without traumatizing the anterior nostrils. So we decided to operate the coanal atresia via the sub-labial method using a trans-nasal dilation catheter (Fig.4) and anterior nostrils were then repaired with z plasty (Fig.5). Following this, beveled edge number 3.5 endotracheal tubes were introduced bilaterally and fixed with sutures sublabially (Fig.6). Post operatively parenteral antibiotics were administered and salin irrigation and suctioning of the tubes were taught to parents. The patient was discharged 3 days post operatively, keeping stents for 4 weeks. Postoperatively her breathing problem was resolved.

## Discussion

Nasal obstruction can lead to compro- mised airways and respiratory distress. Congenital bony nasal deformities are being recognized as an important cause of newborn airway obstruction. The etiologies vary and include choanal atresia, congenital nasal cavity stenosis, pyriform aperture stenosis, nasal agenesis and rarely tumors (3,4). Nasal hypoplasia is seen in many craniofacial syndromes. Apert syndrome often manifests as bilateral narrowing of the bony nasal cavity with choanal stenosis or atresia. Fraser syndrome, a rare autosomal recessive disorder, manifests as cryptophthalmos and nasal anomalies, including a broad nose with midline groove, depressed nasal bridge, hypoplastic nares with colobomas, choanal stenosis, and a beaklike appearance. Binder syndrome, or nasomaxillary hypoplasia, is characterized by mid-face retrusion, hypoplasia of the anterior nasal spine, short columella, reduced naso-frontal angle, high arc palate, half moon shaped nostrils and convex upper lip (5). 

Craniofacial microsomia and Goldenhar syndrome can both affect the nose with varying degrees of maxillary hypoplasia. Apart from syndromic symptoms of nasomaxillary hypoplasia, it usually has sporadic inheritance. Recurrence in sibs with unaffected parents has been repoted seven times and an affected parent and child (or sib-ship) have been noted 10 times (6-8). Although our patient has hypoplastic nostrils with respiratory distress due to bilateral coanal atresia (unrelated to hypoplastic nostrils) and short columella, other features of syndromic hypoplastic mid-face are absent and we didn’t find similar cases in other reports. Furthermore, the patient’s mother had a half moon appearance without a mid-facial retrusion. It seems that this problem solely has no symptoms and signs and it may be problematic in association with other syndromic features. So in combination with, other signs it needs to be repaired surgically such as in association with coanal atresia.

## Conclusion

Nasal obstruction can lead to airway compromise and respiratory distress. Congenital bony nasal deformities are being recognized as an important cause of newborn airway obstruction. Nasal hypoplasia is seen in many craniofacial syndromes. Although our patient had hypoplastic nostrils with respiratory distress due to bilateral coanal atresia, correction of hypoplastic nostrils was necessary for completing the operation of choanal atresia.
